# Anlotinib Combined With Anti-PD-1 Antibodies Therapy in Patients With Advanced Refractory Solid Tumors: A Single-Center, Observational, Prospective Study

**DOI:** 10.3389/fonc.2021.683502

**Published:** 2021-10-07

**Authors:** Min Yuan, Zhongzheng Zhu, Wei Mao, Hui Wang, Hong Qian, Jianguo Wu, Xianling Guo, Qing Xu

**Affiliations:** Department of Oncology, Shanghai Tenth People’s Hospital, Tongji University, Shanghai, China

**Keywords:** anlotinib, anti-PD-1 antibody, angiogenesis, immunotherapy, serum cytokine

## Abstract

**Introduction:**

Anlotinib (AL3818) is a novel multi-target tyrosine kinase inhibitor (TKI) targeting vascular endothelial growth factor receptor (VEGFR) and suppressing tumor growth. Modulation of tumor suppressive immune microenvironment *via* the inhibition of vascular endothelial growth factor may augment the activity of immune checkpoint inhibitors. Here we described the results of safety, and clinical efficacy of anlotinib combined with immunotherapy in patients with advanced solid tumors, the serum cytokine levels, and peripheral blood T lymphocyte populations were detected simultaneously.

**Methods:**

Twenty six cases with advanced late-stage cancers including lung, gallbladder, endometrial, gastric, pancreatic, penile cancers and melanoma were treated since January 2019. Patients received a combination of anlotinib (12mg) once daily on day 1 to day 14 (21 days as a course) plus anti-PD-1 antibodies every 3 weeks until progression or intolerable toxicity. Imaging was performed every 6 weeks for the first year of therapy. Blood samples were collected from patients prospectively. Serum interleukin (IL)-2, IL-4, IL-6, IL-10, Tumor necrosis factor-α (TNF-α), interferon-γ (IFN-γ) and circulating immune cell subsets were measured at baseline and after two cycles of treatment *via* flow cytometry.

**Results:**

There were ten tumor types enrolled with lung, gallbladder, cholangiocarcinoma and soft tissue sarcoma being the most common. Most patients had received front line treatments for metastatic disease (80.8%). The objective response rate (ORR) was 23.1%, including one complete response (CR) (3.8%) and five partial responses (PR) (19.2%) and a disease control rate (DCR=CR+PR+SD) of 80.8% (21 of 26). The median PFS was 4.77 months (95% CI: 4.10-5.44 months). Three patients (11.5%) had grade 3 treatment-related adverse events. There were no grade 4 or 5 treatment-related adverse events. Grades 3 toxicities included hand-foot syndrome (n=2) and hypertension (n=1). Higher serum IL-2, IL-4, IL-10, TNF-α, IFN-γ levels and lower ratios of CD4/CD8 T cells were found in the responders compared with non-responders.

**Conclusions:**

The preliminary data showed that the combination of anlotinib and anti-PD-1 antibodies demonstrated promising durable antitumor efficacy with acceptable toxicity in patients with various advance tumors, and promoted favorable changes in serum IL-2, IL-4, IL-10, TNF-α, IFN-γ levels and circulating immune cell subsets in clinical responders. It is worth to further validate the efficacy in a randomized prospective trial.

## Introduction

Angiogenesis is the process of forming new blood vessels from preexisting blood vessels, and it is also considered as one of the hallmarks of cancer. The overexpression of pro-angiogenic factors can drive the angiogenesis in cancer progression. Growing tumors require new vascular supply to provide essential nutrients and oxygen, which initiating tumor angiogenesis abnormally (1). In addition, low oxygenation concentrations in tissues may trigger tumor angiogenesis, which leads to cancer cells and stromal cells (fibroblasts and macrophages) expressing multiple growth factors *via* the hypoxia-induced factors (HIFs) (2, 3). The hypoxic tumor microenvironment also can promote the upregulation of PD-1 on T cells. It has been reported that the normalization of the tumor vasculature can be remodeled towards appropriate dose of anti-angiogenic treatment, by reducing vascular permeability and interstitial fluid pressure. The normalized tumor vasculature has been proposed to synergize with cancer immunotherapy by reducing tissue hypoxia and enhancing the delivery of cytotoxic agents (4, 5). The hypoxic tumor microenvironment also can promote the upregulation of PD-1 on T cells (6, 7). In addition, there was a significant association between vascular normalization and improved survival in patients treated with chemotherapy (8–10). It has been observed that the use of anti-angiogenic therapy can normalize the tumor vasculature in some preclinical and clinical studies, at least transiently. However, due to the transient and dynamic feature of this process, it remains challenging to improve the effect of immune checkpoint inhibitors by optimizing vascular normalization. The benefits are limited and there is still no biomarker have been found to predict patient’s response (11, 12). Moreover, vessel perfusion can be reduced by inhibiting tumor blood vessel formation excessively, which impedes drug delivery and immune cell infiltration. Patients develop resistance to anti-angiogenic drugs by increased hypoxia, which can also aggravate immunosuppression (13–15). Therefore, our goal for the present study is to evaluate the efficiency and safety for combining anti-angiogenic therapy with an anti–PD-1 agent in patients with advanced refractory solid tumors, and to analyze the potential biomarkers for immune correlates of clinical benefit in this treatment strategy.

Cancer immunotherapy has emerged as an alternative therapeutic approach for various types of cancer over the last decade, including melanoma, NSCLC, urothelial carcinoma and head and neck carcinoma (16–20). It has been shown that blocking PD-1/PD-L1 signaling pathway may normalize the adaptive immune system and reverse an immunosuppression phenotype. When PD-L1 engages PD-1, this triggers exhaustion of CD8+ cytotoxic T cells entered the tumor, and also compromised the response of tumor-reactive cytolytic T cell. Inhibition of the PD-1/PD-L1 pathway can lead to elevation of pro-inflammatory anti-tumor responses such as tumor antigen recognition, proliferation and activation of cytotoxic CD8+ T cells (21, 22). Blockade of the PD-1/PD-L1 pathway using monoclonal antibodies has shown successful results with durable responses and prolonged survival in patients with several solid tumor types. Importantly, the responses are often lasting for years or indefinitely, and without causing serious toxicity in most patients (20, 23, 24).

Thus, the relationships between angiogenesis and immunity are subtle and intricate in tumors. Vascular endothelium plays an important role as a barrier and activates immunity by increasing the expression of endothelial cell adhesion molecules (25). Initial clinical data also support a potentially synergistic effect of blocking angiogenesis and immune checkpoint. In a phase I clinical trial, it was shown that ipilimumab and bevacizumab can increase tumor antigen recognition and infiltration of T cells in melanomas (26). In another clinical study, it was revealed that the combination of atezolizumab and bevacizumab increased the number of intratumoral CD8+ T cells and migration of antigen-specific T cells (27). Combinations of anti-angiogenics with immunotherapy are now being tested in over one hundred phase I/II clinical trials.

Anlotinib is a novel oral multi-target tyrosine kinase inhibitor that can inhibit vascular endothelial growth factor receptor (VEGFR) type 2 and 3, fibroblast growth factor receptor (FGFR) 1-4, platelet-derived growth factor receptor (PDGFR) α and β, stem cell factor receptor (c-Kit), and RET (28). Anlotinib has been approved for refractory advanced NSCLC as a third-line treatment by the China Food and Drug Administration (CFDA) on May 9, 2018 (29). However, few clinical studies have investigated the efficacy and safety of anlotinib combined with immunotherapy in the real world with detailed data. In the present study, we assessed the efficacy and toxicity of anlotinib combined with anti-PD-1 antibodies in advanced solid tumors in clinical practice. We also assessed changes in circulating subsets of immune cells and cytokines to understand treatment response.

## Material and Methods

### Patient Selection

This study was a single-center, observational, prospective study of patients with advanced solid tumors received anlotinib and anti-PD-1 antibodies treatment in Shanghai Tenth People’s Hospital from January 2019 to March 2020. The baseline characteristics and prior lines of treatment were retrospectively analyzed. The clinical stages of all patients were classified based on the eighth edition of the American Joint Committee on Cancer (AJCC). The Eastern Cooperative Oncology Group (ECOG) PS was evaluated prior to treatment. All data were obtained from follow-up visits and medical records. All patients gave their written informed consent for biobanking, use of biomaterials and clinical data for scientific purposes. This study was approved by the Ethics Committee of Shanghai Tenth People’s Hospital.

### Treatment

All patients agreed to use anlotinib combined with anti-PD-1 antibodies. The specific regimen was: anlotinib 8-12mg q.d. for 2 consecutive weeks with 1 week of rest. Patients received one of the following anti-PD-1 agents every 3 weeks until disease progression, clinical deterioration, or unacceptable toxicity: sintilimab (Innovent Biologics, China), toripalimab (Shangha Merck & Co.), camrelizumab (Jiangsu Hengrui Medicine, China), nivolumab (Bristol-Myers Squibb, USA) or pembrolizumab (Merck & Co., USA). All patients were evaluated for toxicity. Adverse events were retrospectively classified according to the Common Terminology Criteria for Adverse Events version 4.0 (CTC4.0). Patients with grade 1 or 2 AEs were advised to continue medication, with follow-up observations in the outpatient department. Patients with grade 3 or higher AEs were suggested to stop the medication and were hospitalized for symptomatic treatment until the grade of AEs was ≤2. The medication was continued if tolerated by the patient, and the medication was stopped if the patient’s safety was at risk or if serious sequelae were suspected.

### Assessment

Tumor assessment was performed according to the Response Evaluation Criteria in Solid Tumors (RECIST version 1.1) (30). Short-term efficacy was chosen to evaluate the treatment effect, which is usually defined as 2 cycles after the combined therapy is established. Clinical responses were categorized as either complete response (CR), partial response (PR), stable disease (SD), or progressive disease (PD). The overall response rate (ORR) corresponds to all cases with CR and PR. The disease control rate (DCR) is defined as the percentage of patients with CR, PR and SD. We refer to the patients with disease control (CR+PR+SD) as the responder group, and patients with PD as the nonresponder group. Progression-free survival (PFS) was calculated as the time from the initiation of treatment with anti-PD-1 combined with anlotinib to PD or death. In cases of failure of the regimen, patients were advised to enter a clinical trial or to start treatment with other drugs. Therefore, it is not objective to assess the efficacy of this regimen based on the overall survival (OS) of patients, and no analysis or discussion was made.

### Cytokine Assays and Flow Cytometry

Blood samples were collected into EDTA-containing collection tubes from each patient within 3 days before the beginning of therapy as baseline, and before the 3rd cycle as post-treatment. Blood samples were centrifuged within 4 h of collection at 2000-4000 rpm for 20 min. The supernatant was collected as plasma and analyzed within 24h.

Samples were analyzed with FACS Canto II flow cytometer manufactured by Becton Dickinson (BD), USA. Measurement of serum interleukin-2 (IL-2), interleukin-4 (IL-4), interleukin-6 (IL-6), interleukin-8 (IL-8), interleukin-10 (IL-10), tumor necrosis factor-α (TNF-α), interferon-γ (IFN-γ), and lymphocyte subsets was assessed by flow cytometry analysis according to the manufacturer’s instructions. Cytokines were performed using Cytokine Combination detection Kit 12-plex by CellGene, China. Immunophenotyping of PBMCs was performed using BD Multitest 6-Color TBNK Reagent. Commercial conjugated antibodies used include CD3 FITC (BD Pharmingen clone SK7), CD16 PE (BD Pharmingen clone B73.1), CD56 PE (BD Pharmingen clone NCAM16.2), CD45 PerCP-Cy5.5 (BD Pharmingen clone 2D1), CD4 PE-Cy7 (BD Pharmingen clone SK3), CD19 APC (BD Pharmingen clone SJ25C1) and CD8 APC-Cy7 (BD Pharmingen clone SK1) antibodies.

### Statistical Analysis

Descriptive statistics (percentages, means, medians, and standard deviations) were used to describe baseline characteristics and clinical features of the sample of patients. The short-term efficacy (ORR) was analyzed by the Chi-square test and Fisher exact test. PFS were analyzed by the Kaplan–Meier method, and subgroups were compared with the log-rank test for the total number of patients. P<0.05 was considered statistically significant. Statistical analyses were performed by SPSS statistical software (version 20.0; SPSS, IBM Corporation).

## Results

### Characteristics of Patients at Baseline

From January 2019 to March 2020, 26 patients were enrolled in this real-world study. In the final eligible sample, 13 (50.0%) were men and 13 (50.0%) were women, and the median age was 66 years (range, 37-77 years). Notably, 53.8% of patients were 65 years or older. 25 patients (96.2%) had ECOG status of 0-1 and 1 (3.8%) had ECOG status of 2. There were ten tumor types enrolled with lung, gallbladder, cholangiocarcinoma and soft tissue sarcoma being the most common (**Table 1** **)**. 14 patients (53.8%) were diagnosed with adenocarcinoma type, 4 (15.4%) with squamous type, and 3 (11.5%) with sarcoma type. 5 patients (19.2%) received anlotinib as first-line therapy, 13 (50.0%) as second-line treatment and 8 (30.8%) as third-line and above therapy. 9 patients received 12 mg anlotinib treatment, 14 received 10 mg anlotinib treatment and 3 received 8 mg anlotinib treatment as starting doses. As for the anti-PD-1 treatment, the numbers of patients received sintilimab, camrelizumab, toripalimab, nivolumab or pembrolizumab treatment were 15 (57.7%), 5 (19.2%), 3 (11.5%), 2 (7.7%) and 1 (3.8%), respectively. Baseline demographics and clinical characteristics are displayed in **Table 2**.

**Table 1:** Tumor types across 26 patients.

Tumor types	patients *N*	ORR	DCR	PFS (month)
lung cancer	9	11.1%	77.8%	4.29
soft tissue sarcoma	4	25.0%	100.0%	5.64
cholangiocarcinoma	4	25.0%	100.0%	6.01
gastric cancer	2			5.10
endometrial carcinoma	2			4.95
tongue cancer	1			11.3
penile cancer	1			7.33
pancreatic cancer	1			1.61
laryngeal cancer	1			1.67
bladder cancer	1			1.70

ECOG, Eastern Cooperative Oncology Group.

**Table 2:** Demographics and baseline characteristics of patients.

characteristics	patients *N*	% of total
Age (years)		
<65	12	46.2
≥65	14	53.8
Gender		
Male	13	50.0
Female	13	50.0
ECOG		
0	16	61.5
1	9	34.6
2	1	3.8
Histological subtype		
Adenocarcinoma	14	53.8
Squamous cell carcinoma	4	15.4
Sarcoma	3	11.5
Other	5	19.2
Metastasis site		
Liver	8	30.8
Bone	8	30.8
Brain	3	11.5
Lines of therapy		
1	5	19.2
2	13	50.0
≥3	8	30.8
Receiving anlotinib dose		
12mg	9	34.6
10mg	14	53.8
8mg	3	11.5
Anti-PD-1 antibodies		
Sintilimab	15	57.7
Camrelizumab	5	19.2
Triprilimab	3	11.5
Nivolumab	2	7.7
Pembrolizumab	1	3.8

ECOG, Eastern Cooperative Oncology Group.

### Efficacy and Survival

**Figure 1:**
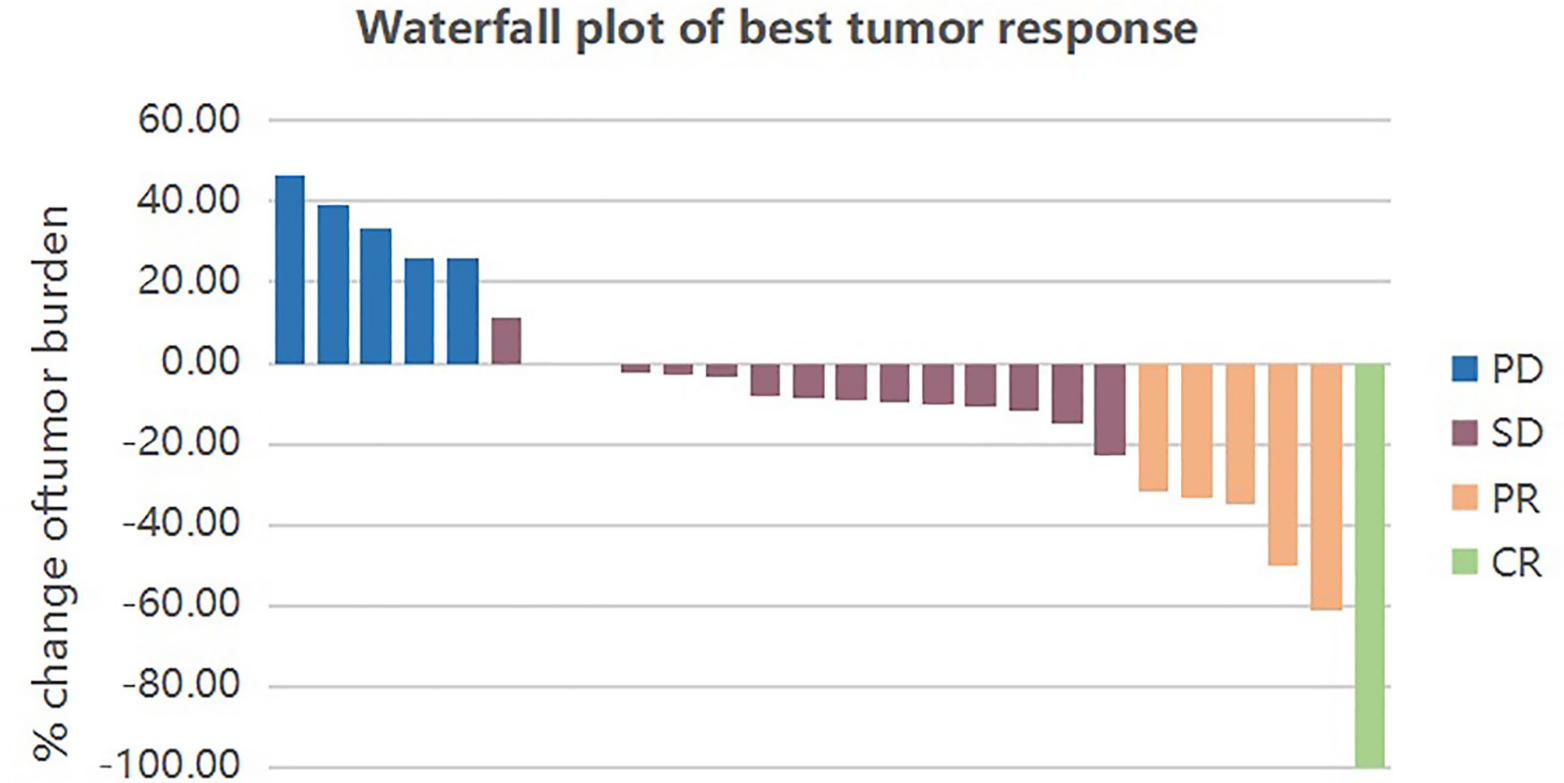
Waterfall plot of best tumor response by RECIST1.1. Each bar represents the response of an individual patient. CR, complete response; PR, partial response; SD, stable disease; PD, progressive disease.

**Figure 2:**
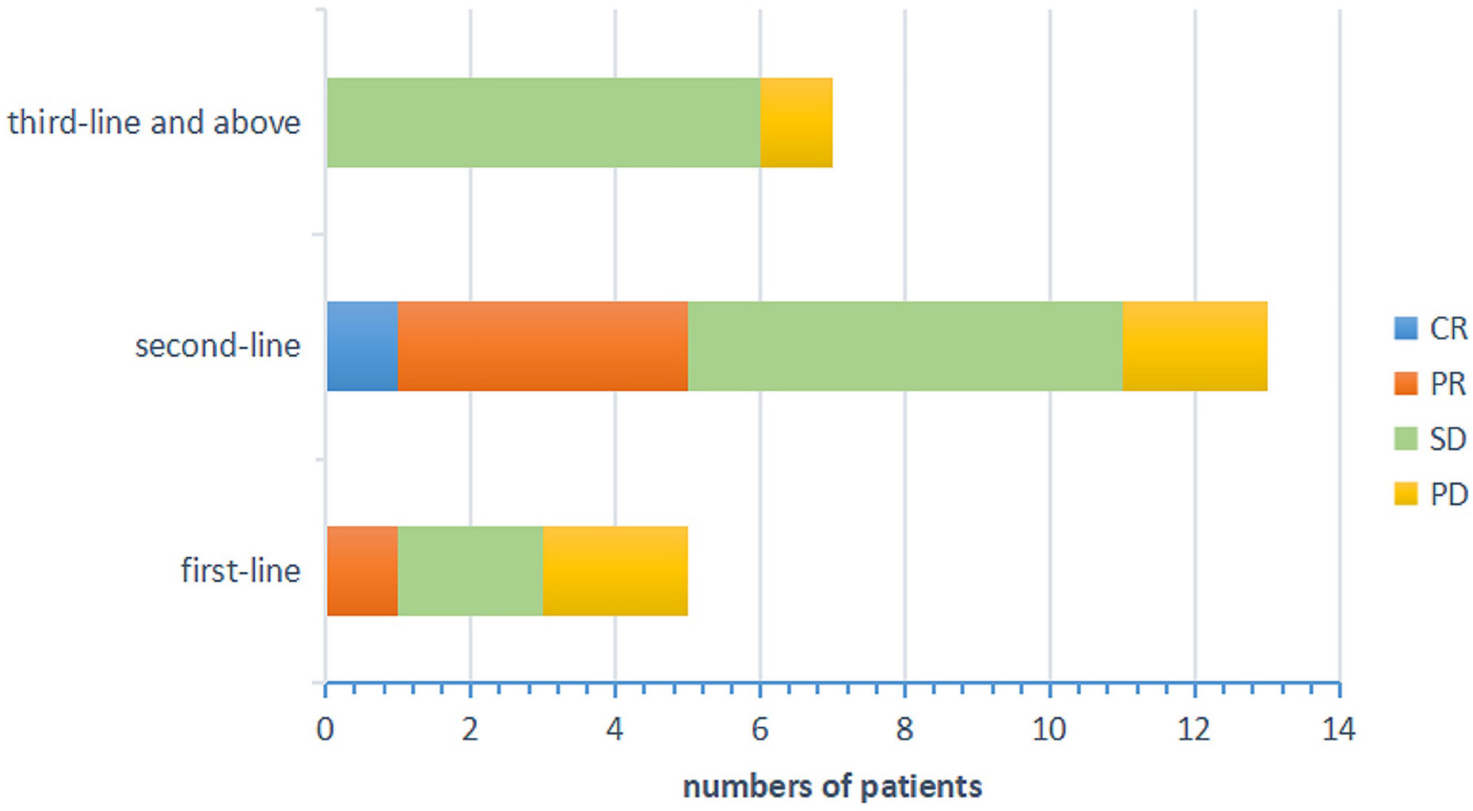
Efficacy evaluation of each treatment groups. CR, complete response; PR, partial response; SD, stable disease; PD, progressive disease.

The primary objective of this clinical trial was to estimate the efficacy, as measured by ORR and DCR. The ORR of 26 patients was 23.08% (3.85% CR and 19.23% PR). The DCR was 80.77% (3.85% CR, 19.23% PR and 57.69% SD) as shown in **Figure 1**. The patients treated with the combination regimen as first-line, second-line and third-line and above treatment were 19.23%, 50.00% and 30.77%. DCR were 60.00% (3/5), 84.62% (11/13) and 87.50% (7/8) in different lines of treatment. The subgroup analysis revealed no statistically significant difference in the DCR of patients receiving different lines of treatment, respectively (p=0.354). The efficacy evaluation of each treatment group is shown in **Figure 2**. Moreover, we analyzed efficacy in several subgroups of common metastatic sites, including liver, bone and brain. The patients with liver metastasis showed an ORR of 37.5% (3/8) and DCR of 87.5% (7/8).

**Figure 3:**
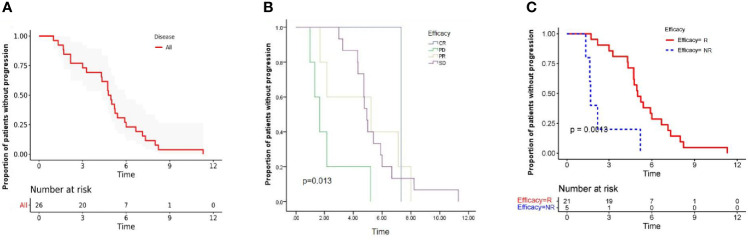
Kaplan-Meier estimates of progression-free survival in all patients **(A)**, and stratified by the efficacy **(B)**. Progression free survival was significantly lower among responder group than non-responder group **(C)**, Long rank test was performed. P value is shown in the graphs. CR, complete response; PR, partial response; SD, stable disease; PD, progressive disease; R, responder; NR, non-responder.

After a median follow-up period of 9.0 months (range, 4.97-20.43 months), 8 patients had died, and no patients remained at the progression-free stage. The median PFS of all patients was 4.77 months (95% CI: 4.10-5.44 months) (**Figure 3A**), CR group was 7.33 months, PR group was 5.23 (95% CI: 0.94-11.80 months), SD group was 4.95 (95% CI: 4.61-5.29 months), PD group was 1.67 (95% CI: 0.91-2.40 months). The difference in PFS was statistically significant, P=0.013 (**Table 3** and **Figure 3B**). In order to identify patients who might receive greater clinical benefits from anlotinib combined with anti-PD1 antibodies, the short-term efficacy of CR or PR or SD is defined as responder group and PD as non-responder group among patients treated with this pattern. Patients experiencing greater short-term efficacy exhibited a significantly longer PFS of 5.00 months (95% CI: 4.31-5.69 months) *vs* 1.67 months for non-responder group (95% CI: 0.94-2.40 months). The difference in PFS was statistically significant, *P*=0.001 (**Figure 3C**). There was also significant difference in PFS associated with ECOG, *P*=0.001. While the median OS was not reached.

**Table 3:** Univariate analysis of correlation between clinical characteristics and median progression-free survival (PFS).

Characteristics	*N*	Median PFS (mo)	95% CI	*P*
Age (years)				0.800
<65	12	4.30	2.50-6.10	
≥65	14	5.20	4.78-5.62	
Gender				0.506
Male	13	5.23	4.70-5.76	
Female	13	4.30	2.78-5.82	
ECOG				0.001
0	16	5.20	3.34-7.06	
1	9	4.73	1.18-8.28	
2	1	1.00		
Histological subtype				0.909
Adenocarcinoma	14	5.00	3.35-6.65	
Squamous cell carcinoma	4	4.73	1.52-7.94	
Sarcoma	3	4.77	4.07-5.47	
Other	5	4.73	0.00-10.23	
Metastasis site				0.999
Liver	8	4.73	3.48-5.98	
Bone	8	4.30	2.22-6.38	
Brain	3	4.30	0.89-7.71	
Lines of therapy				0.317
1	5	4.33	0.00-10.77	
2	13	4.95	2.13-7.77	
≥3	8	4.77	4.40-5.14	
Efficacy				0.013
CR	1	7.33		
PR	5	5.23	0.94-11.80	
SD	15	4.95	4.61-5.29	
PD	5	1.67	0.91-2.40	

### Safety

Overall, 46.15% patients experienced treatment-related AEs, as shown in **Table 3**. Nine grade 1-2 side effects (34.62%) and three grade 3 events (11.54%) but no grade 4 or 5 events were observed. The most common adverse effects were fatigue 5 (19.23%), decreased appetite 4 (15.38%), and hand-foot syndrome 4 (15.38%), which did not seem to have much effect on the progress of treatment. The most serious adverse event was grade-3 hand-foot syndrome and hypertension, which occurred in 3 patients (11.54%) (**Table 4**). That may be associated with the use of anti-angiogenic agents. In brief, no patients discontinued treatment by treatment-related AEs in our study, which indicates that anti-PD-1 antibodies combined with anlotinib therapy is tolerable in the real world.

**Table 4:** Toxicity profile and safety summary.

Treatment-related adverse events	Grade1-2, *n* (%)	Grade3-5, *n* (%)
Fatigue	5 (19.23%)	0
Decreased appetite	4	0
Hand-foot syndrome	2	2
Hypertension	1	1
Diarrhea	2	0
Hypothyroidism	1	0
hematuria	1	0

### T Cell Subsets and Cytokines

**Figure 4:**
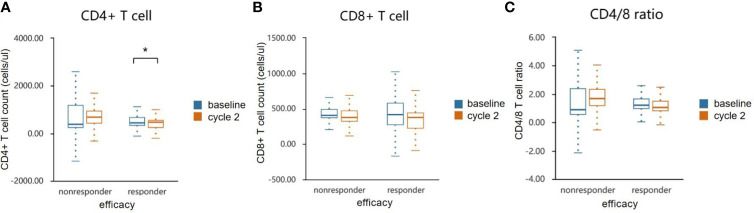
Effects of combination therapy on circulating lymphocyte population cells in the responder and non-responder groups. The absolute numbers of CD 4+ **(A)**, CD 8+ T cells **(B)** and CD4/8 ratio **(C)** were calculated at baseline and after two cycles treatment. Wilcoxon paired t-test was performed to detect the statistical significance (*P < 0.05).

PBMC collected before and after two cycles of treatment were analyzed for levels of CD4+ and CD8+ T-cells shown in **Figure 4**. Compared with the non-responder group, anlotinib combined with anti-PD-1 antibodies caused a significant decline of CD4+ T-cells in blood (p<0.05, **Figure 4A** and **Table 5**), decrease of CD8+ T cells were observed in both groups (**Figure 4B**). The ratios of CD4+/CD8+ T cells in the responder group were decreased, but the results were reversed in the nonresponder group (**Figure 4C**). However, changers of CD8+ T cells and CD4/8 ratios between two groups showed no significant difference.

**Table 5:** Summary of phenotypic and cytokines analyses of patients.

	Efficiency	Baseline	Cycle2	*p*
CD4+ cells	R	596.9	422.9	0.041
	NR	520.8	682.4	0.604
CD8+ cells	R	451.1	399.3	0.429
	NR	372.8	359.2	0.816
CD4/8 ratio	R	1.56	1.14	0.177
	NR	1.30	1.99	0.284
IL-2	R	1.19	2.70	0.207
	NR	2.54	1.62	0.473
IL-4	R	2.15	2.73	0.702
	NR	3.08	1.68	0.256
IL-6	R	19.04	22.04	0.272
	NR	17.80	32.89	0.262
IL-10	R	5.19	5.83	0.613
	NR	3.10	2.16	0.457
TNF-α	R	1.98	2.97	0.482
	NR	5.46	2.99	0.454
IFN-γ	R	3.33	5.51	0.474
	NR	2.65	1.90	0.742

**Figure 5:**
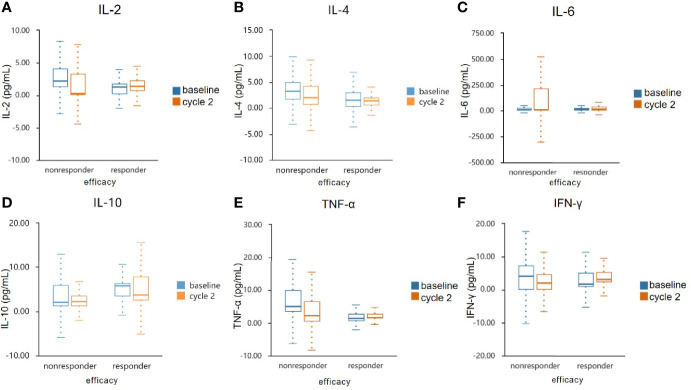
Serum cytokine levels in the responder and non-responder groups. The serum specimens of IL-2 **(A)**, IL-4 **(B)**, IL-6 **(C)**, IL-10 **(D)**, TNF-α **(E)**, and IFN-γ **(F)** were measured obtained at baseline and after two cycles treatment. IL, interleukin; TNF-α, tumour necrosis factor α; IFN-γ, interferon γ.

We measured IL-2, IL-4, IL-6, IL-10, TNF-α, and IFN-γin serum specimens obtained before and after two cycles of treatments. There were numerical increases of IL-2, IL-4, TNF-α, and IFN-γafter treatment in patients with disease control (CR + PR + SD) but decreases in patients with progressive disease. IL-10 was decreased in responder group but increased in nonresponder group. IL-6 was increased in both groups (**Figure 5** and **Table 5**). However, cytokines showed no significant difference. This may be due to the sample size was too small to reach a significant difference.

## Discussion

The ability of inducing angiogenesis is critical to the development of cancer. The formation of new blood vessels prevents the tumor from being deprived of oxygen and nutrients, which is typical of cancer cells. Hypoxia-driven vascular endothelial growth factor (VEGF), a major regulator of angiogenesis, stimulates the proangiogenic signaling pathway by binding to VEGF receptor 2 (VEGFR2). The hypoxia caused by vessel abnormality further induces resistance to other therapies and creates an immunosuppressive microenvironment (4). Studies in recent years have demonstrated that the key to successful anti-angiogenic therapy is to control the dose to a vessel normalizing level (4, 31). Blood vessels within solid tumors are often morphologically abnormal and functionally impaired, resulting in reduced infiltration of immune effector cells into the tumor (32). In addition, tumor blood vessels have impaired perfusion capacity, which creates increased intratumoral hypoxia that inhibits the activity of infiltrated cytotoxic T cells and promotes the accumulation of suppressive immune cells such as myeloid-derived suppressor cells (MDSC) and regulatory T cells (33). Studies have shown that VEGF could reprogram the immunosuppressive microenvironment through a variety of mechanisms, such as promoting immunosuppressive cytokines (IL-10, TGFβ), enhancing expression of inhibitory checkpoints (PD1, CTLA4, and LAG-3) in CD8+ T cells, and increasing the presence of MDSCs and Tregs (34, 35). Thus, antiangiogenics could potentially improve immunotherapy effectiveness by normalizing the tumor microenvironment. This mechanism was confirmed in a pre-clinical study, which suggested that the application of anti-VEGF-A antibody (sunitinib) in CT26 tumor-bearing mice increased the infiltration of cytotoxic tumor-infiltrating lymphocytes (TIL) and decreased expression of PD-1 in CD8+T cells (36). Moreover, another research provided evidence in reverse that anti-PD-L1 therapy can make tumors sensitive to antiangiogenic therapy and improve its efficacy (37).

Numerous studies in various malignancies have proved the efficacy of combining PD-1/PD-L1 inhibitors and anti-VEGF agents. Combination therapy with pembrolizumab and axitinib was approved by the FDA for treatment-naïve patients with metastatic renal cell carcinoma (RCC) (38). In hepatocellular carcinoma (HCC), a randomized phase III clinical trial, IMBRAVE 150 (NCT03434379), demonstrated significant improvements in co-primary end points, PFS and OS, using the combination of atezolizumab (anti-PD-L1) and bevacizumab (anti-VEGF-A) compared with sorafenib (39). This study evaluated the clinical benefits of anlotinib in combination with anti-PD-1 antibodies in the real-world treatment of patients with advanced solid tumors. The results demonstrate that, combination of anti-PD-1 antibody and anlotinib has efficacy against advanced solid tumors in later-line treatment and patients can tolerate it. Our results demonstrated the efficacy of anti-PD-1 plus anlotinib, as shown by the ORR of 23.08% and DCR of 80.77%, with a median PFS of 4.77 months (95% CI: 4.10-5.44 months). Interestingly, there has also been shown therapeutic benefit in patients with liver metastases, which is considered as a common metastatic site and a negative prognostic indicator (40–42). In our study, only two patients (7.69%) underwent dose reduction but no one discontinued therapy, which suggested that the combination therapy was well tolerated.

The limitation of immunotherapy in solid tumors is the activation of multiple immunosuppressive components in the tumor microenvironment (43). VEGF plays an immuno-suppressive role in the TME by accumulating Tregs and repolarizing tumor-associated macrophages (TAMs) to M2-like phenotypes (44). VEGF also induces the TOX-mediated exhaustion program in CD8+ CTLs (45). However, CD8+ CTLs play a critical role in suppressing tumor angiogenesis by secreting IFN-γ (46). IFN-γ directly inhibits the proliferation and migration of human endothelial cells and restrains the proliferation of endothelial cells and tumor vascularization (47, 48). Hyperactive IFN-γ/STAT1 signaling promotes M1-like TAM reprogramming, leading to vascular remodeling and consequent tumor eradication (49, 50). In this study, we focused on the analysis of changes in circulating immune populations and cytokines after starting treatment of anlotinib combined with anti-PD-1 antibodies. We observed an elevated level of IL-2, IL-4, TNF-α, and IFN-γin responders, and decrease of IL-10 in responders. However, the differences between the two groups have not reached statistical significance, likely due to the small number of enrolled patients. Significant dynamic changes were observed for CD4+ T cell counts. The ratio of CD4+/CD8+ post-treatment was declined in the responders, which might be either the result of better vessel normalization, or the better immunotherapy response.

However, we found that there are several limitations in our study. The first is the limited number of subjects to recruit, the sample size is too small to reach a significant difference. Second, we only analyzed serum CD4+ and CD8+ T cells, although immune cells may play different roles and prognoses in the tumor microenvironment. Third, as a real world study, it is possible that patients had potential systemic inflammation unrelated to cancer which may affect the T cell subsets and cytokines in blood. The finding of an immune signature with an easily reproducible and robust flow cytometry assessment in peripheral blood could greatly facilitate patient selection in the clinic. Thus, further studies of this aspect are required to characterize their potential role as a biomarker, and also investigate whether patients have an OS benefit.

## Conclusion

This study demonstrated that the combination of anlotinib and anti-PD-1 antibodies demonstrated promising durable antitumor efficacy with acceptable toxicity in patients with various advance tumors. The reciprocal effects of anti-angiogenic therapy and immunotherapy makes combining these two modalities a promising novel treatment combination that warrants further investigation in both preclinical and clinical settings. Favorable changes in serum cytokine levels and circulating immune cell subsets seem to be crucial for clinical benefit in the treatment. Noninvasive peripheral blood analysis of some markers could be distinctly helpful in assessing the immunity status in the tumor microenvironment.

## Data Availability Statement

All data included in this study are available upon request by contact with the corresponding authors. 

## Ethics Statement

The studies involving human participants were reviewed and approved by Ethics Committee of Shanghai Tenth People’s Hospital. The patients/participants provided their written informed consent to participate in this study.

## Author Contributions

MY worked for guarantor of integrity of entire study and statistical analysis. QX worked for study concepts and design. XG worked for manuscript editing and revision. WM, ZZ, HW, JW, and HQ worked for clinical studies and data acquisition and analysis. All authors contributed to the article and approved the submitted version.

## Funding

This study was funded by National Natural Science Foundation of China (No. 81772905); Western Medicine Guiding Project of Shanghai Science and Technology Commission (No.17411967300); Key Project of Shanghai Health and Family Planning Commission (No.201640020).

## Conflict of Interest

The authors declare that the research was conducted in the absence of any commercial or financial relationships that could be construed as a potential conflict of interest.

## Publisher’s Note

All claims expressed in this article are solely those of the authors and do not necessarily represent those of their affiliated organizations, or those of the publisher, the editors and the reviewers. Any product that may be evaluated in this article, or claim that may be made by its manufacturer, is not guaranteed or endorsed by the publisher.
