# Corrigendum: Anlotinib Combined With Anti-PD-1 Antibodies Therapy in Patients With Advanced Refractory Solid Tumors: A Single-Center, Observational, Prospective Study

**DOI:** 10.3389/fonc.2021.796625

**Published:** 2022-01-04

**Authors:** Min Yuan, Zhongzheng Zhu, Wei Mao, Hui Wang, Hong Qian, Jianguo Wu, Xianling Guo, Qing Xu

**Affiliations:** Department of Oncology, Shanghai Tenth People’s Hospital, Tongji University, Shanghai, China

**Keywords:** anlotinib, anti-PD-1 antibody, angiogenesis, immunotherapy, serum cytokine

In the original article, there was an error. The median PFS was incorrectly written as 8.37 months (95% CI: 6.5-10.0 months) in the abstract.

A correction has been made to Abstract, Results, Paragraph 1:

The median PFS was 4.77 months (95% CI: 4.10-5.44 months).

The authors apologize for this error and state that this does not change the scientific conclusions of the article in any way. The original article has been updated.

## Publisher’s Note

All claims expressed in this article are solely those of the authors and do not necessarily represent those of their affiliated organizations, or those of the publisher, the editors and the reviewers. Any product that may be evaluated in this article, or claim that may be made by its manufacturer, is not guaranteed or endorsed by the publisher.

